# Copper and Antimicrobial Residues in the Liver and Kidney—Antimicrobial Resistance and Cu Tolerance Unrelated in *Escherichia coli* from Piglets’ Faeces

**DOI:** 10.3390/microorganisms12122553

**Published:** 2024-12-11

**Authors:** Maria Manuel Donato, Olga Cardoso, Gabriela Assis, Sara Carolina Henriques, Andreia Freitas, Fernando Ramos

**Affiliations:** 1 Centro de Investigação em Meio Ambiente, Genética e Oncologia (CIMAGO), Faculdade de Medicina, Universidade de Coimbra, Azinhaga de Santa Comba, 3000-548 Coimbra, Portugal; mariamanueldonato@gmail.com; 2 Department of Chemical Engineering, Chemical Engineering and Renewable Resources for Sustainability (CERES), Faculdade de Farmácia, Azinhaga de Santa Comba, Universidade de Coimbra, 3000-548 Coimbra, Portugal; 3 Laboratório de Controlo da Alimentação Animal, Unidade Estratégica de Investigação e Serviços, Tecnologia e Segurança Alimentar, Instituto Nacional de Investigação Agrária e Veterinária, I.P, Av. da República, Quinta do Marquês, 2780-157 Oeiras, Portugal; gabriela.assis@iniav.pt (G.A.); andreia.freitas@iniav.pt (A.F.); 4 Research Institute for Medicines (iMed.ULisboa), Faculty of Pharmacy, Universidade de Lisboa, 1649-003 Lisboa, Portugal; saracarolinahenriques@gmail.com; 5 Laboratório Nacional de Referência para a Segurança Alimentar, Instituto Nacional de Investigação Agrária e Veterinária, I.P., Rua dos Lágidos, Lugar da Madalena, Vairão, 4485-655 Vila do Conde, Portugal; 6 Rede de Química e Tecnologia/Laboratório Associado para a Química Verde (REQUIMTE/LAQV), Rua Dom Manuel II, Apartado 55142, 4051-401 Porto, Portugal; framos@ff.uc.pt

**Keywords:** piglets, copper, antimicrobial residues, *Escherichia coli*, metals and antimicrobial susceptibility, One Health

## Abstract

Antimicrobials, widely used in livestock, have induced the emergence of antimicrobial-resistant bacteria, prompting farmers to explore alternatives like copper. This study aims to determine antimicrobial residues and Cu concentrations in the liver and kidney of piglets and to investigate the correlation between Cu and antimicrobial use and the resistance to Cu and antimicrobials of *Escherichia coli* isolated from piglets’ faeces. Antimicrobial residues were quantified by UHPLC-ToF-MS; Cu was quantified using FAAS; microbiological methods were used for *E. coli* isolation, CuSO_4_ minimal inhibitory concentration (MIC), and antimicrobial susceptibility; and to detect genes, Real-Time PCR was used. Cu concentrations and antimicrobial residues in piglet livers and kidneys revealed no significant differences. Antimicrobial residues were detected in a significant number of livers and kidneys. While Cu concentrations in the liver were within adequate ranges, those in the kidney exceeded the recommended levels. *E. coli* isolates from piglet faeces exhibited high antimicrobial drug resistance (AMR), with no clear link to Cu exposure. The genes *cop*A, *pco*A, and *pco*D, associated with Cu tolerance, were predominantly found in isolates with a CuSO_4_ MIC of 8 mM. Cu was not used excessively, suggesting that Cu did not replace antimicrobials. *E. coli* was mostly resistant to antimicrobials and it was not possible to demonstrate that Cu was the trigger for this resistance. There was no relationship between Cu tolerance and AMR in *E. coli* isolates. This study highlights the need for further research on the complex interplay between metals, antimicrobials, and bacterial resistance in livestock, impacting ‘One Health’.

## 1. Introduction

Antimicrobial agents have been extensively utilized worldwide to treat livestock diseases, such as post-weaning diarrhoea in pigs, which pose a significant threat to the swine industry. They were also commonly used as growth promoters. However, the overuse of antimicrobials has led to the emergence of highly resistant bacteria [1,2,3]. In 2006, the European Union (EU) banned the use of antimicrobials as growth promoters [4]. As an alternative, heavy metal ions such as copper have been proposed to enhance animal health and promote growth rates [5]. The mechanisms behind this growth-promoting effect are still not fully understood. One explanation is attributed to an antimicrobial effect leaving more nutrients and energy available to the pig itself [6,7,8]. The maximum Cu concentration allowed in animal feed in EU legislation is 170 mg Cu/kg compound diet for piglets (<12 weeks) and 25–30 mg Cu/kg compound diet for all other categories [9].

Cu is an essential trace element for every life form and is an important structural component or regulatory cofactor of many different enzymes in several important biochemical pathways in plants and animals. Organisms have evolved homeostatic abilities that allow for control over inner concentrations of trace elements, maintaining optimal levels despite a fluctuating external availability [10,11,12].

Swine tolerates high levels of dietary Cu and begins to accumulate Cu in the liver at high Cu intake levels (up to 50 times the dietary requirement). The liver is the main reservoir of Cu and the main mediator of its homeostasis; under normal exposure conditions, the Cu absorbed in the body is regulated by the hepatobiliary pathway and 98% is excreted in the faeces, with the remainder lost via urine [9,12].

The actual concentration of metals used in feed samples is significantly higher than the animals’ growth requirements. An excessive and inappropriate use of metals has resulted in their excretion in the animals’ faeces and their accumulation in the environment, leading to the co-selection of antimicrobial-resistant bacteria [2]. There has been great concern about heavy metals indirectly selecting for antimicrobial drug resistance (AMR) in bacteria due to a coupling of the resistance mechanisms against heavy metals and antimicrobials. Cross-resistance is a physiological mechanism that provides tolerance to more than one antimicrobial agent and heavy metal (e.g., multidrug efflux pumps that extrude antimicrobials and heavy metals); another mechanism is co-resistance, when two or more genetically linked resistance genes in bacteria are located next to each other on one mobile genetic element, such as plasmids, integrons, and genetic islands [2,10,13,14]. Resistance mechanisms to both antimicrobials and metals are issues recognized in the ‘One Health’ approach.

The use of indicator organisms from the intestinal microbiota of animals can be a useful tool for evaluating the impact of antimicrobial and metal usage in pig farming. *Escherichia coli* is a commonly used indicator organism due to its commensal nature and abundance in the animal gut. *E. coli* is known to acquire resistance to antimicrobials, and these resistance patterns are considered to represent the majority of resistance traits found in Gram-negative bacteria in animals [3]. Bacteria have developed various strategies to cope with the toxic effects of Cu in cells. Since Cu often enters the bacterial cell in an unspecific manner (utilizing other metal uptake systems), bacteria cannot limit the amount of Cu that enters the cytoplasm. Therefore, they have to develop mechanisms to expel excess cytoplasmic Cu. In Gram-negative bacteria, the best-characterized system for expelling excess Cu from the cytoplasm is the ATPase CopA, encoded by the chromosomal *cop*A gene [15]. In *E. coli*, in addition to the chromosomal mechanism, Cu resistance is also mediated through a plasmid-borne Cu (*pco*) resistance cluster consisting of seven *pco*ABCDRSE genes encoding the following: PcoA, a periplasmic multicopper oxidase; two periplasmic proteins, PcoC and PcoE; the outer membrane protein PcoB; and the inner membrane protein PcoD. The expression of *pco*ABCD is regulated by PcoRS, while the expression of *pco*E is regulated via a chromosomally encoded system, CusRS [15,16].

In Europe, as reported by the EFSA [8,9], studies about the complex interactions between metallic ions, specially Cu, antimicrobial residues, and bacterial resistance and tolerance to ions in piglets are scarce and not conclusive, as is the case in Portugal, a member country of the European Union.

To provide knowledge of the Portuguese reality on this subject, the first aim of this study is to ascertain whether there is a relationship between the use of Cu and antimicrobials by determining the concentrations of Cu and the antimicrobial residues in the liver and kidney of piglets. The second aim is to assess whether there is a correlation between the usage of these agents and the resistance to Cu (determination of CuSO_4_ MIC and genes related to Cu tolerance) and antimicrobials in *E. coli* obtained from piglet faeces, as an indicator of the gut microbiota.

## 2. Materials and Methods

### 2.1. Sampling and E. coli Isolation

In this study, faeces (*n* = 60), liver (*n* = 56), and kidney (*n* = 60) samples were collected from randomly selected healthy piglets (*n* = 60; weighing 5–8 kg) between October 2018 and May 2019. The piglets came from an abattoir in Mealhada, which receives piglets from different regions of Portugal due to the high demand for roasting piglets (‘leitão à bairrada’), a local gastronomic specialty [17]. Sampling was carried out under a veterinarian’s supervision.

The samples collected from each animal consisted of one whole kidney; 200 g of the liver (right lobe); and a minimum of 10 g of faeces. All samples were individually placed in sterile plastic bags and immediately transported to the laboratory. Liver and kidney samples were stored at −18 °C until analysis.

The isolation of *E. coli* was performed on the day of collection as described elsewhere using Lauryl Sulphate Agar [17].

### 2.2. Cu Quantification and Allocation of Levels in the Liver and Kidney

The quantification of Cu in liver and kidney samples was obtained by FAAS using an air-acetylene flame after dry-ashing following an internal method based on ISO 14082:2003 [18] and ISO 6869:2000 [19]. A Thermo Scientific iCE 3000 single-element hollow cathode lamp spectrometer (Lisbon, Portugal) was used, emitting light at 324.8 nm [20].

Cu content was obtained from a calibration curve using Cu standard solutions in hydrochloric acid according to a similar procedure referred elsewhere [20]. Each sample was analysed in duplicate. The final result was calculated as the mean value of the two measurements obtained.

Cu levels in the liver were classified by López-Alonso [21] as deficient (0.3–1.02 mg/kg), marginal (4–7 mg/kg), adequate (7–25 mg/kg), high (25–200 mg/kg), and toxic (150–15,000 mg/kg).

The same study [21] also defined five levels of Cu concentration in the kidneys: deficient (2–4 mg/kg), marginal (4–7 mg/kg), adequate (7–10 mg/kg), high (12–25 mg/kg), and toxic (30–1200 mg/kg).

### 2.3. Quantification of Antimicrobial Residues

UHPLC-ToF-MS was used to determine 43 antimicrobials from seven therapeutic classes. Antimicrobial and internal standards of ≥98% purity were purchased from Sigma-Aldrich (Madrid, Spain). The extraction procedure and UHPLC-ToF-MS conditions have been described previously [22].

### 2.4. Antimicrobial and CuSO_4_ Susceptibility Assays

Minimum inhibitory concentrations (MICs) for CuSO_4_, defined as the lowest CuSO_4_ concentration at which no visible growth was observed, were determined by agar dilution using standard bacteriological methods [20]. Metallic stock solution (CuSO_4_ × 5H_2_O) was added to molten Mueller–Hinton (MH) agar to achieve final concentrations between 0.125 and 16 mM for CuSO_4_ [23,24,25], and the pH was adjusted to 7.2. Each assay was carried out in triplicate. Plates with no Cu present were used as a control. 

Isolates were tested for antimicrobial susceptibility by agar disc diffusion on MH agar following the European Committee on Antimicrobial Susceptibility Testing (2020) guidelines [26]. The antimicrobials (Liofilchem^®^s.r.l., Roseto degli Abruzzi, Italy) used included the following seven classes of beta-lactams: amoxicillin–clavulanic acid (AMC) (20–10 µg); piperacillin (PIP) (30 µg); cefoxitin (FOX) (30 µg); ceftazidime (CAZ) (10 µg); cefepime (FEP) (30 µg); imipenem (IP) (10 µg); and aztreonam (AZT) (30 µg). The remaining 3 antimicrobials were from other families and included sulfamethoxazole–trimethoprim (SXT) (23.75–1.25 µg); ciprofloxacin (CIP) (5 µg); and amikacin (AK) (30 µg). *E. coli* ATCC 25922 was used as a control.

The definition of multidrug resistance (MDR) adopted in this study is the one of the European Centre for Prevention and Disease Control (ECDC) published by Magiorakos [27]. MDR is understood as resistance to at least one antimicrobial in three or more antimicrobial classes; extensive drug resistance (XDR) as resistance to at least one antimicrobial in all but two or fewer antimicrobial classes; and pan drug resistance (PDR) as resistance to all agents in all antimicrobial classes.

### 2.5. Determination of Cu Tolerance Genes

Cu tolerance genes were determined by Real-Time PCR (LightCycler, Roche Diagnostics, Germany) on crude bacterium DNA (Table 1).

PCR was performed in a volume of 20 µL with 4.0 µL LightCycler FastStart DNA MasterPLUS SYBR Green I^®^ (Roche Diagnostics, Mannheim, Germany). In all cases, 45 amplification cycles were performed after an initial denaturation cycle at 95 °C for 10 min. The specific conditions for denaturation, annealing, and extension for each set of primers are shown in Table 1. Melting curves were also automatically plotted and analysed using LightCycler software v.1.5. PCR products were detected on 2% agarose gels stained with ethidium bromide. The gels were visualized by UV light. By comparing the two results, it was possible to establish specific melting temperatures for the identification of each gene. Positive (*E. coli* 31.3 of this study) and negative (*E. coli* 36.6 of this study) controls were always included. To avoid methodological errors, all procedures were repeated by another technician who was blinded to the previous results.

### 2.6. Statistical Analysis

Liver and kidney samples were categorized according to the presence or absence of antimicrobial residues.

Quantified Cu concentrations were summarized by mean and 95% confidence interval (CI) of the mean.

Welch’s two-sample *t*-test was performed to compare Cu concentration in the liver and kidney with the absence and presence of antimicrobial residues. Similarly, Welch’s two-sample t-test was used to detect differences in Cu concentration in the liver and kidney as a function of *E. coli* identified in faeces as S or MDR.

The chi-squared test was used to test for differences in the proportion of Cu MIC as a function of the *E. coli* identified as S or MDR.

The binomial test was used to compare the observed frequencies of the presence and absence of genes associated with bacterial tolerance to Cu in *E. coli* identified as MDR.

All results were evaluated at a 5% significance level.

Statistical and graphical analyses were performed in R software version 4.2.1 (R Foundation for Scientific Computing, Vienna, Austria, 2013).

## 3. Results

The quantification of Cu and antimicrobial residues (Table S1) was determined in the livers of 56 piglets and in the kidneys of 60 piglets.

The determination of Cu concentration in the liver prompted the grouping of samples by the different levels described in the Materials and Methods section: three samples (5.4%) were allocated at a high level; twenty-eight (50%) at an adequate level; seven (12.5%) at a deficient level; and eighteen (32.1%) at a marginal level.

Regarding antimicrobial residues in the liver, 32 samples (57.1%) did not show any residues, 18 (32.1%) had one or two residues, and 6 (10.7%) had three or more residues. One objective of this study was to relate the presence or absence of these residues to the Cu concentration, hence; in the analysis of the results, only the presence or absence of antimicrobial residues detected in the liver was considered.

In the livers of 32 piglets with no antimicrobial residues, the mean Cu concentration was 10.7 mg/kg (95% CI: [7.5–13.8] mg/kg), a value within the adequate level. An adequate level of Cu was observed in sixteen liver samples; a high level of Cu was observed in two livers; a deficient level of Cu was observed in four livers; and a marginal level of Cu was observed in ten livers. In the remaining 24 livers where antimicrobial residues were present, the mean Cu concentration was 10.1 mg/kg (95% CI: [6.7–13.5] mg/kg), a value within the adequate level. The Cu levels in these livers were similar to the levels observed in the samples where no residues of antimicrobials were detected: an adequate level of Cu was observed in twelve livers; a high level of Cu was observed in one liver; a deficient level of Cu was observed in three livers; and a marginal level of Cu was observed in eight livers.

**Figure 1:**
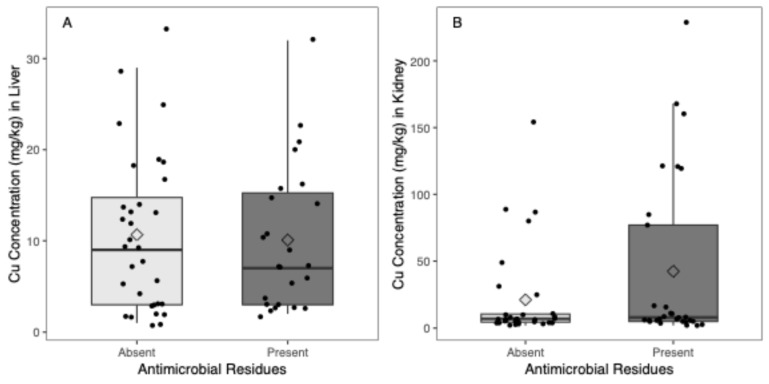
Box plot of Cu concentration by the absence or presence of antimicrobial residues detected in the (**A**) liver and (**B**) kidney.

Figure 1A presents the distribution of Cu concentration by the absence or presence of antimicrobial residues found in the liver in the form of box plots. Individual values are presented as dots, and the mean values for each group are represented as diamond shapes.

In piglets’ livers, no significant differences in Cu concentration were found with the absence or presence of antimicrobial residues (0.6 [−3.9–5.1], *p*-value = 0.798) (Figure 1A).

The quantification of Cu was performed in 60 piglets’ kidneys and the samples were grouped in the different levels described in the Materials and Methods section: 14 kidneys (23.3%) presented a toxic level; 5 kidneys (8.3%) presented a high level; 12 kidneys (20%) presented an adequate level; 21 kidneys (35%) presented a deficient level; and 8 kidneys (13.3%) presented a marginal level.

Regarding antimicrobial residues in the kidney, 31 kidneys (51.7%) presented no residues, with a mean Cu concentration of 21.1 mg/kg (95% CI: [8.3–34] mg/kg), a value within the high level. Five kidneys presented a marginal level; ten kidneys presented a deficient level; eight kidneys presented an adequate level; two kidneys presented a high level; and six kidneys presented a toxic level.

The remaining 29 kidneys (48.3%) presented at least one antimicrobial residue, with a mean Cu concentration of 42.4 mg/kg (95% CI: [18.3–66.5] mg/kg), a value within the toxic level. Three kidneys presented a marginal level; eleven kidneys presented a deficient level; four kidneys presented an adequate level; three kidneys presented a high level; and eight kidneys presented a toxic level.

Figure 1B presents the distribution of Cu concentration by the absence or presence of antimicrobial residues found in the kidney in the form of box plots. Individual values are presented as dots, and the mean values for each group are represented as diamond shapes.

In piglets’ kidneys, no significant differences in Cu concentration were found with the absence or the presence of antimicrobial residues (−21.3 [−48.1–5.6], *p*-value = 0.118) (Figure 1B).

From the piglets’ faeces, 276 isolates of *E. coli* were isolated. Their susceptibility to the antimicrobials used in human health was determined to observe the resistance of these isolates. The highest resistance was determined for amoxicillin and clavulanic acid (74.3%), followed by piperacillin (65.2%); trimethoprim and sulfamethoxazole (58.3%); ciprofloxacin (41.7%); and amikacin (39.9%). The other beta-lactams used in this study showed resistance rates of less than 20%, while 3.3% of the isolates were resistant to imipenem. According to the results obtained for susceptibility to antimicrobials, MDR was observed in 180 (65%) *E. coli* isolates, and 96 (35%) were considered susceptible (S) to the majority of the antimicrobials tested [20].

**Figure 2:**
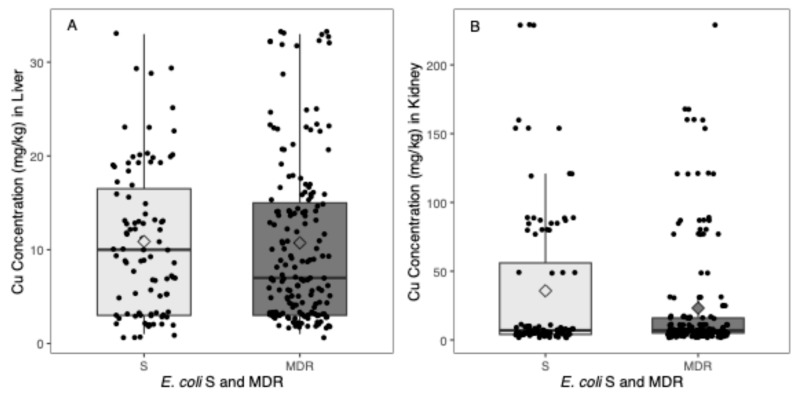
Box plot of the distribution of Cu concentration in the (**A**) liver and (**B**) kidney as a function of the *E. coli* identified as S or MDR.

A total of 260 *E. coli* isolates (56 piglets’ faeces) were correlated with Cu concentrations in the liver (*n* = 56). Of these, 91 (35.0%) *E. coli* isolates were classified as S, and the mean Cu concentration in the liver in these cases was 10.9 mg/kg (95% CI: [9.3–12.5] mg/kg); 169 (65.0%) were MDR, and the mean of Cu concentration in these cases was 10.7 mg/kg (95% CI: [9.4–12.1] mg/kg) (Figure 2A).

Figure 2A presents the distribution of Cu concentration in the liver as a function of the *E. coli* identified as S or MDR in the form of box plots. Individual values are presented as dots, and the mean values for each group are represented as diamond shapes.

No significant differences in Cu concentration in the liver were found between *E. coli* classified as S or MDR (0.1 [−1.9–2.2], *p*-value = 0.89) (Figure 2A).

A total of 276 *E. coli* isolates (60 piglets’ faeces) were correlated with Cu concentration in the kidney (*n* = 60). A total of 96 (34.8%) *E. coli* were identified as S, and the mean of Cu concentration in the kidney was 35.8 mg/kg (95% CI: [24.6–46.9] mg/kg) in these cases; 180 (65.2%) were considered MDR, and in these cases, the mean Cu concentration was 23.2 mg/kg (95% CI: [17.2–29.1] mg/kg) (Figure 2A).

Figure 2B presents the distribution of Cu concentration in the kidney as a function of the *E. coli* identified as S or MDR in the form of box plots. Individual values are presented as dots, and the mean values for each group are represented as diamond shapes.

In the kidney, a significant decrease in Cu concentration of 12.6 mg/kg (95% CI: [0–25.2] mg/kg) was found in MDR *E. coli* when compared with S *E. coli* (*p*-value = 0.05) (Figure 2B).

**Figure 3:**
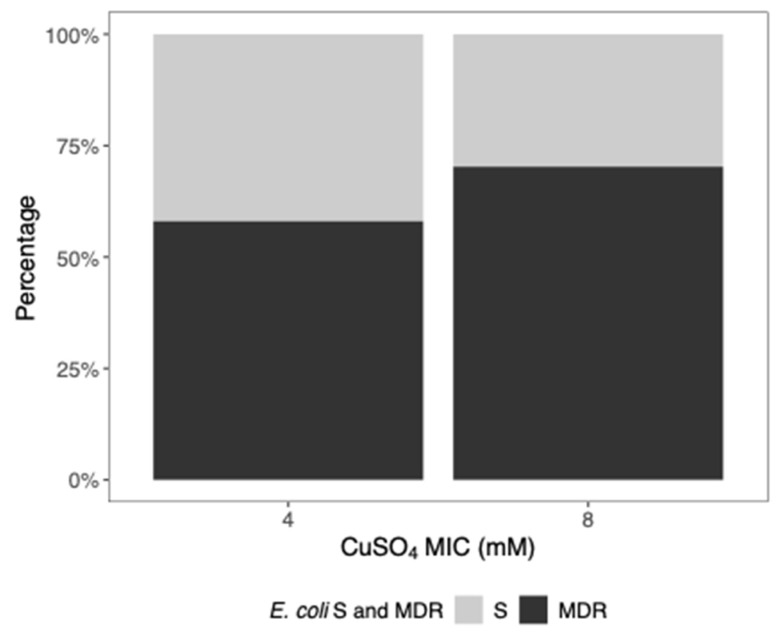
Percentage of S and MDR *E. coli* as a function of CuSO_4_ MIC.

Regarding the CuSO_4_ MIC, 159 (57.6%) isolates had a MIC of 8 mM and 117 (42.4%) had a MIC of 4 mM. A total of 69.8% of *E. coli* isolates with a MIC of 8 mM were MDR and 30.2% were S; 59.0% of *E. coli* isolates with a MIC of 4 mM were MDR and 41.0% were S (Figure 3). No statistical differences were found between *E. coli* categorized as S and MDR as a function of CuSO_4_ MIC (*p*-value = 0.0618).

The *pco*A, *pco*D, and *cop*A genes, which are associated with bacterial tolerance to Cu, were detected by Real-Time PCR. The *pco*A gene was observed in 122 (44%) of the isolates, the *pco*D gene in 118 (43%), and the *cop*A gene in 209 (76%) of the isolates. *E. coli* with the *cop*A gene was more likely to have a CuSO_4_ MIC of 8 mM [123 (58.9%)] than a CuSO_4_ MIC of 4 mM [86 (41.1%)], (*p*-value = 0.0126); isolates with the *pco*A gene were more likely to have a CuSO_4_ MIC of 8 mM [84 (68.9%)] than a CuSO_4_ MIC of 4 mM [38 (31.1%)] (*p*-value < 0.0001); and when the *pco*D gene was present, the bacteria were also more likely to have a CuSO_4_ MIC of 8 mM [80 (67.8%)] than a CuSO_4_ MIC of 4 mM [38 (32.2%)], (*p*-value < 0.0001). These last two genes were simultaneously present in 71 isolates, and these *E. coli* isolates were more likely to have a CuSO_4_ MIC of 8 mM [50 (70.4%)] than a CuSO_4_ MIC of 4 mM [21 (29.6%)], (*p*-value < 0.0001).

The presence of the three genes was observed in 54 (20%) of the isolates. These *E. coli* isolates had a higher probability of having a CuSO_4_ MIC of 8 mM [38 (70%)] than of having a CuSO_4_ MIC of 4 mM [16 (30%)] (*p*-value = 0.0038).

## 4. Discussion

In the liver and kidney of piglets, no statistically significant differences were found between Cu concentration and the presence or absence of antimicrobial residues. Therefore, it appears that Cu was not replacing antimicrobials or vice versa. Although antimicrobials were being used, since they were detected in 42.9% of the livers and 48.3% of the kidneys tested, despite being forbidden as growth promoters in the EU [4], antimicrobials can still be used to treat animal infections. Studies on antimicrobial residues and Cu accumulation in piglets’ kidneys and livers that could be compared to the present work were not found in the literature.

The average concentration of Cu detected in the piglets’ liver was at an adequate level. A similar result was obtained by López-Alonso [21]. As it is known, when Cu intake is greater than 50× the amount required, Cu accumulates in the liver [9,12]. The results of this study essentially demonstrated adequate levels of this ion in the liver; therefore, it appears that the piglets’ diet was not supplemented with surplus Cu. Regarding the Cu concentrations in the kidney, it was found that the average concentration was higher, including high and toxic levels, and greater than that reported by other authors [21]. This result could be explained by the excessive administration of Zn [20], as corroborated by other authors suggesting that high dietary Zn feeding in pigs leads to Cu co-accumulation in the kidney, and there is yet no clear explanation for why co-accumulation occurs only in the kidney and not in other organs [28,29,30].

*E. coli* isolates were used as an indicator organism of the piglets’ microbiota to observe resistance to the metals and antimicrobials used for clinical and farming purposes. Other bacteria, such as enterococci, could be used as indicator microorganisms [31]. Therefore, *E. coli* is a good indicator microorganism because it is a natural and specific inhabitant of the intestines of warm-blooded animals, including humans, that can be easily and quickly detected using standard microbiological methods. The *E. coli* isolates in this study were more resistant to antimicrobials than those reported in previous studies on countries in the EU, including Portugal [32,33,34]. Similar results of high resistance levels in *E. coli* from farm animals have been reported in many countries worldwide [33,35,36,37], and a recent study showed that 72.5% of *E. coli* isolated from fresh livestock faeces in China was MDR [38]. In a surveillance report from the ECDC and WHO [39], *E. coli* from humans in Portugal was more susceptible to various classes of antimicrobials than the isolates from this study. A previous work by the authors [17] demonstrated that these isolates were resistant to antimicrobials and presented different genes for resistance to beta-lactams, a great class of antibiotics with extensive human clinical uses.

One of the limitations of this study is that Cu concentration was not determined in the faeces. It is known that hepatobiliary excretion is the main regulatory pathway of adequate Cu concentrations and that Cu is excreted mainly through the faeces [9,10,11,12]. In this study, *E. coli* was isolated from faeces, and it can be inferred that it was challenged by Cu from this route of excretion.

As the mean concentration of Cu in the liver was at an adequate level, the amount of Cu in the faeces would not have been high either, and the *E. coli* would not have been stressed by this ion; also, no significant differences in Cu concentration were found between *E. coli* considered S or MDR (*p* = 0.89).

A significant decrease in Cu concentration was found in MDR *E. coli* compared to S *E. coli* (*p* = 0.05) in the kidney, although a positive correlation between Cu concentration and AMR has been observed in other matrices, such as manure [40]. The susceptibility profile observed in the *E. coli* isolates might be not linked with the high concentration of Cu in the kidney, since they were isolated from faeces and not from urine.

However, the high percentage of MDR *E. coli* (65%) seems to be related to antimicrobial exposure (antimicrobial residues were present in approximately 45% of the viscera) or the transfer of resistance determinants from other bacteria [13,14,17], and it may not be linked to the concentration of Cu in the faeces, since Cu is expected to have been at adequate levels, as previously stated.

In this study, there was no relationship between Cu tolerance and AMR in *E. coli* isolates, which is in line with other studies [5,37,41]. Despite this, several studies have reported an association between Cu tolerance and AMR [2,10,13,38,40], including in other bacterial genera, like enterococci [31]. Therefore, among the Cu-tolerant *E. coli* isolates (CuSO_4_ MIC of 8 mM), 69.8% was MDR, a result similar to that presented by Tan et al. [38]. In a previous study, the authors demonstrated a similar behaviour of *E. coli* isolates in terms of Zn tolerance and AMR, although the concentrations of Zn in the viscera (high and toxic levels of Zn were predominant) were higher than the concentration of Cu in the same matrices [20].

This study was designed to verify the presence of the *cop*A, *pco*A, and *pco*D genes in *E. coli*, as well as whether there was a relationship between them and phenotypic tolerance to Cu (MIC determination). The presence of the *cop*A gene in 76% of *E. coli* isolates was in line with current knowledge, because this chromosomal gene is ubiquitous and is the central component of Cu homeostasis [15,42]; also, *E. coli* with the *cop*A gene was more likely to be resistant to Cu (58.9% had a CuSO_4_ MIC of 8 mM). *E. coli* with the *pco*A and/or *pco*D genes was also more resistant to Cu (70.4% had a CuSO_4_ MIC of 8 mM). Tan et al. [38] reported that the Cu resistance genes *pco*ABCDRS were detected in 15% of *E. coli* isolates from faeces, and in this study, the simultaneous presence of these two genes was 25%. These genes were described in a plasmid-borne Cu system associated with Cu efflux and might spread the metal resistance that is often co-selected with AMR [15,38].

## 5. Conclusions

In this study, it appears that Cu was not overused and did not replace antimicrobials in feed additives as growth promoters. Cu and antimicrobials were not interlinked.

There was no relationship between Cu tolerance and AMR in *E. coli* isolates.

The presence of genes related to Cu efflux was present in the *E. coli* isolates more resistant to Cu (CuSO_4_ MIC of 8 mM).

*E. coli* isolates were mostly resistant to antimicrobials, and it was not possible to demonstrate that Cu was the trigger for this resistance.

In conclusion, the accumulation of Cu and antimicrobial residues in the livers and kidneys of piglets, and particularly their implication in *E. coli* resistance, presents significant challenges for food safety and public health. Addressing these issues requires a multifaceted approach that includes better management practices in livestock production, stringent regulatory oversight, and ongoing research into the mechanisms of resistance and the impact of heavy metals on microbial populations, with implications for ‘One Health’.

## Figures and Tables

**Table 1:** Specific primers and PCR conditions used in amplification of Cu tolerance genes.

Gene	Primer Sequence (5′–3′)	PCR Conditions	Product Length (bp)	Reference
*cop*A	AAC GGT TTC CGC ACA TTG CCG ATG CTG TTG TAG ATA AAG G	95 °C, 10 s; 55 °C, 5 s; 72 °C, 25 s	623	[24]
*pco*A	TTG TCT GAC TGG ACC GAT G TTA TCC GTT TCT GAC GCA G	95 °C, 10 s; 53 °C, 5 s; 72 °C, 32 s	797	[24]
*pco*D	CAG GAA CGG TGA TTG TTG TA CCG TAA AAT CAA AGG GCT TA	95 °C, 10 s; 53 °C, 5 s; 72 °C, 28 s	700	[16]

## Data Availability

Data is contained within the article or Supplementary Materials.

## Supplementary Materials

The following supporting information can be downloaded at https://www.mdpi.com/article/10.3390/microorganisms12122553/s1, Table S1: Cu concentration and antibiotic residues detected in piglets’ kidneys and livers.
