# Supplementary material for: Copper and Antimicrobial Residues in the Liver and Kidney—Antimicrobial Resistance and Cu Tolerance Unrelated in Escherichia coli from Piglets’ Faeces
Source: Microorganisms. 2024 Dec 11;12(12):2553. doi: 10.3390/microorganisms12122553 (PMC11677510; doi:10.3390/microorganisms12122553)
Supplement: Supplementary file 1 [file microorganisms-12-02553-s001.zip › microorganisms-3242766-supplementary.pdf]

Table S1: Cu concentration and antibiotics residues detected in kidney and liver piglets

Piglet Number	Antibiotic Residue	
	Kidney	Liver
16	Tilmicosin	Doxycycline
17	Tilmicosin	
18		
19	Tilmicosin;Sulfisomidine	
20	Tilmicosin	
21	Doxycycline	Doxycycline
22		Enrofloxacin
23		Doxycycline
24		
25		
26	Doxycycline;Sulfachloropyridazine;Sulfadiazine; Tilmicosin	Doxycycline; Tilmicosin;Trimethoprim
27	Doxycycline;Sulfachloropyridazine;Sulfadiazine; Tilmicosin	Doxycycline; Sulfachloropyridazine;Sulfadiazine; Tilmicosin
28	Doxycycline; Tilmicosin	Doxycycline; Tilmicosin;Trimethoprim
29	Doxycycline;Sulfachloropyridazine;Sulfadiazine; Tilmicosin	Doxycycline;Trimethoprim
30		
31		
32	Trimethoprim	
33	Trimethoprim	
34	Trimethoprim	
35	Trimethoprim	
36		
37		
38		
39		
40		
41		Enrofloxacin
42		Enrofloxacin; Ciprofloxacin
43		Enrofloxacin; Ciprofloxacin
44	Enrofloxacin	Enrofloxacin; Ciprofloxacin
45		
46		Enrofloxacin; Ciprofloxacin; Danofloxacin; Spiramicyn

47	Enrofloxacin; Ciprofloxacin	Enrofloxacin; Ciprofloxacin
48		Enrofloxacin
49		Enrofloxacin; Ciprofloxacin; Danofloxacin; Spiramicyn
50	Enrofloxacin	Enrofloxacin
51	Enrofloxacin	Enrofloxacin; Ciprofloxacin
52	Enrofloxacin	Enrofloxacin; Ciprofloxacin
53	Enrofloxacin	Enrofloxacin
54	Enrofloxacin	Enrofloxacin; Ciprofloxacin
55		
56		
57	Oxytetracyclin	
58		
59	Oxytetracyclin	
60	Enrofloxacin	Enrofloxacin
61		
62	Enrofloxacin	Enrofloxacin; Ciprofloxacin; Danofloxacin
63	Enrofloxacin; Danofloxacin	Enrofloxacin
64		Danofloxacin
65		
66		
67		Danofloxacin
68	Sulfadiazine; Trimethoprim	
69	Sulfadiazine; Trimethoprim	
70	Sulfadiazine; Trimethoprim	
71	Sulfadiazine	
72		
73		
74		
75		

Nd – not determined
